# Heuristics for multiobjective multiple sequence alignment

**DOI:** 10.1186/s12938-016-0184-z

**Published:** 2016-07-15

**Authors:** Maryam Abbasi, Luís Paquete, Francisco B. Pereira

**Affiliations:** 1 CISUC, Department of Informatics Engineering, University of Coimbra, Polo II, Pinhal de Marrocos, 3030-290 Coimbra, Portugal; 2 Polytechnic Institute of Coimbra, Rua Pedro Nunes, Quinta da Nora, 3030-199 Coimbra, Portugal

**Keywords:** Multiple sequence alignment, Iterated local search, Multiobjective optimization

## Abstract

**Background:**

Aligning multiple sequences arises in many tasks in Bioinformatics. However, the alignments produced by the current software packages are highly dependent on the parameters setting, such as the relative importance of opening gaps with respect to the increase of similarity. Choosing only one parameter setting may provide an undesirable bias in further steps of the analysis and give too simplistic interpretations. In this work, we reformulate multiple sequence alignment from a multiobjective point of view. The goal is to generate several sequence alignments that represent a trade-off between maximizing the substitution score and minimizing the number of indels/gaps in the sum-of-pairs score function. This trade-off gives to the practitioner further information about the similarity of the sequences, from which she could analyse and choose the most plausible alignment.

**Methods:**

We introduce several heuristic approaches, based on local search procedures, that compute a set of sequence alignments, which are representative of the trade-off between the two objectives (substitution score and indels). Several algorithm design options are discussed and analysed, with particular emphasis on the influence of the starting alignment and neighborhood search definitions on the overall performance. A perturbation technique is proposed to improve the local search, which provides a wide range of high-quality alignments.

**Results and conclusions:**

The proposed approach is tested experimentally on a wide range of instances. We performed several experiments with sequences obtained from the benchmark database BAliBASE 3.0. To evaluate the quality of the results, we calculate the hypervolume indicator of the set of score vectors returned by the algorithms. The results obtained allow us to identify reasonably good choices of parameters for our approach. Further, we compared our method in terms of *correctly aligned pairs* ratio and *columns correctly aligned ratio* with respect to reference alignments. Experimental results show that our approaches can obtain better results than TCoffee and Clustal Omega in terms of the first ratio.

## Background

Multiple sequence alignment (MSA) is of central importance to bioinformatics. This technique is useful to compare new sequences with other genomic sequences, unveiling their shared information and their significant differences. MSA methods are mainly essential to analyse biological sequences and to design applications in structure modeling, functional prediction, phylogenetic analysis and sequence database searching [1]. Currently, these approaches are also used in applications, for example, to compare protein structures, to predict protein mutations and interactions or to reconstruct phylogenetic trees [2, 3]. Moreover, these tools have found their place in medicine as well, mainly in the context of genetic screening and genetic engineering [4].

Most of the known alignment approaches have diverse optimization functions, along with assorted heuristics to search for the optimum alignment. These techniques consider a weighted sum formulation that maximizes the substitution score and penalizes indels/gaps. This is the usual procedure even in the case of pairwise sequence alignment [5]. However, the way of setting up the weights is very often not trivial. Moreover, analyzing only one alignment may lead to too simplistic interpretations of the data [6].

A multiobjective formulation of sequence alignment provides the practitioner a set of alignments that represents the trade-off between decreasing the number of gaps and increasing similarity. In bioinformatics, this formulation and algorithms can be found already for pairwise sequence (DNA/Protein) alignment  [7–10]. Abbasi et al. [7] present dynamic programming algorithms to compute the optimal set of alignments by treating the number of indels/gaps and the scores for (mis)matches/substitution as separate objectives. They also apply this method to analyze the construction of phylogenetic trees. Taneda [11] describes a heuristic approach for pairwise RNA sequence alignment that incorporates RNA structure information to approximate a set of optimal alignments. Schnattinger et al. [12] extend the work of Taneda by computing the optimal set. They treat the sequence alignment and the consensus structure calculation as separate objectives and solve both problems simultaneously with a dynamic programming approach. An extensive review about other problems in bioinformatics that are formulated as multiobjective optimization problems is explained in Handl et al. [13].

Few work has been done on multiobjective MSA (MMSA), which is much harder to be solved from a computational point of view. Only very recently, MSA has been treated as multiobjective optimization problem. Ortuño et al.  [14] used a multiobjective evolutionary algorithm based on the NSGA-II to optimize sum-of-pairs score, total columns and number of gaps. In another article [15], the authors extended the previous work by applying further biological features and considering different objectives such as strike score, non-gaps percentage and totally conserved columns. Likewise, Soto et al.  [16] applied a multiobjective evolutionary algorithm to optimize pre-aligned sequences by considering entropy and the metric Metal.

In this article, we propose to approach MMSA with heuristic methods, extending the formulation given in Abbasi et al. [7] for an arbitrary number of sequences. The approach considers the *sum-of-pairs* score vector by maximizing the substitution score, based on a substitution matrix, and minimizing the number of indels/gaps. This article is an extended version of a conference article [17] providing a more thorough experimental analysis of the algorithms proposed.

## Methods

We first give a definition of multiple sequence alignment, followed by its multiobjective counterpart. Then, a local search strategy is proposed.

### Multiple sequence alignment

In MSA, homogeneous characters of a group of sequences are aligned together in columns. The following definition introduces the problem of MSA in a more mathematical form [18].

#### **Definition 1**

*Multiple sequence alignment.* Let $A_1 = (a_{1,1}, \ldots , a_{1,n_1} ), \ldots , A_m = (a_{m,1} ,\ldots , a_{m,n_m})$ be *m* strings over an alphabet $\Sigma$. Let $'-' \not \in \Sigma$ be an indel symbol, let $\Sigma ^\prime = \Sigma \cup \{-\}$ and let $\lambda$ denote the empty string. Let $h : (\Sigma ^\prime )^* \mapsto \Sigma ^*$ be a homomorphism defined by $h(a) = a$ for all $a \in \Sigma$, and $h(-) = \lambda$. A multiple sequence alignment $\phi$ of $(A_1 , \ldots , A_m )$ is a *m*-tuple of $B_1= (b_{1,1}, \ldots , b_{1,\ell }), \ldots , B_m = (b_{m,1}, \ldots , b_{m,\ell })$ of strings of length $\ell \ge \max \{A_i |1 \le i \le m\}$ over the alphabet $\Sigma ^\prime$, such that the following conditions are satisfied:

(i) $$|B_1 |= |B_2 |= \ldots = |B_m |$$
(ii) $h(B_i ) = A_i$ for all $i \in \{1, \ldots , m\}$
(iii) For all $j \in \{1, \ldots , \ell \}$ there exists an $i \in \{1, \ldots , m\}$ such that $b_{i,j} \ne '-'$.

A way of evaluating the quality of an alignment $\phi$ is to score its columns by the *sum-of-pairs* score function. The score of a column $j=1,\ldots ,\ell$ is defined as:

$$\begin{aligned} S(j) = \sum _{1\le i < k \le m} sc(b_{i,j}, b_{k,j}) \end{aligned}$$

where the score $sc(b_{i,j}, b_{k,j})$, for $b_{i,j}, b_{k,j} \in \Sigma$, comes from a substitution matrix used for scoring pairwise sequence alignments (such as PAM and BLOSUM). Indels are scored by defining $sc('-', \cdot ) = sc(\cdot ,'-') = W_d$, where $W_d$ is the weight of an indel, and $sc('-','-') = 0$. The score for alignment $\phi$ is computed as

$$\begin{aligned} SP(\phi ) = \sum _{j=1}^\ell S(j) \end{aligned}$$

### Multiobjective multiple sequence alignment

Many problems of practical relevance are frequently characterized by different objectives that simultaneously have to be taken into account when it comes to solve the problem. In most cases, these objectives conflict with each other and optimizing the problem for a specific objective might compromise other objectives. An appropriate approach to a multiobjective problem is to obtain a set of solutions in such a way that each of which cannot be improved in one objective without deteriorating at least one of the others [19].

Traditionally, approaches for solving sequence alignment are performed with a single objective function, which is based on a weighted sum of matches, mismatches, insertions and deletions. However, there is no agreement on how to specify weights for these parameters. In a multiobjective formulation, the practitioner does not have to define weights and can get access to much more information [10].

Let $\phi$ be an alignment of *m* sequences $(A_1,\ldots ,A_m)$, as defined in the previous section. We define the following two score functions

$$\begin{aligned} S_s(j) = \sum _{1\le i< k \le m} s(b_{i,j}, b_{k,j}) \quad \text{ and } \quad S_d(j) = \sum _{1\le i < k \le m} d(b_{i,j}, b_{k,j}) \end{aligned}$$

where the score $s(b_{i,j}, b_{k,j})$, for $b_{i,j}, b_{k,j} \in \Sigma$ is obtained from a substitution matrix and $d(b_{i,j}, b_{k,j})$, for $b_{i,j}, b_{k,j} \in \Sigma ^\prime$, is 1 if either $b_{i,j}= {\text{'}}-{\text{'}}$ or $b_{k,j}= {\text{'}}-{\text{'}}$, and 0, otherwise. The multiobjective score sum-of-pairs for alignment $\phi$ is

$$\begin{aligned} BSP(\phi ) = \left( BSP_s(\phi ) = \sum _{j=1}^\ell S_s(j),BSP_d(\phi ) = \sum _{j=1}^\ell S_d(j)\right) \end{aligned}$$

Given two alignments $\phi$ and $\phi ^\prime$, $BSP(\phi ) \ge BSP(\phi ^\prime )$ ($\phi$ dominates $\phi ^\prime$) if and only if it holds that $BSP_s(\phi ) \ge BSP_s(\phi ^\prime )$ and $BSP_d(\phi ) \le BSP_d(\phi ^\prime )$, with at least one strict inequality. An alignment $\phi ^*$ is *Pareto optimal* if there exists no other alignment $\phi$ such that $BSP(\phi ) \ge BSP(\phi ^*)$. The set of all Pareto optimal alignments is called *Pareto optimal alignment set*. The image of a Pareto optimal alignment in the score space is a *non-dominated score* and the set of all non-dominated scores is called *non-dominated score set*.

Although pairwise sequence alignment can be solved efficiently, that is, the running time to find the non-dominated score set is a polynomial function of the size of the sequences, this is no longer the case for the multiple counterpart for an arbitrary number of sequences. Thus, the goal, in practice, is to find an approximation to the non-dominated score set in a reasonable amount of time. In this article we explore the application of heuristic methods, in particular, local search algorithms.

### Pareto local search

A local search algorithm starts from a feasible solution and searches *locally* for better neighbors to replace the current one. This neighborhood search is repeated until no improvement is found anymore and the algorithm stops in a local optimum. In the context of MMSA, the neighborhood function associates a set of feasible alignments $N(\phi )$ to every feasible alignment $\phi$. An alignment $\phi$ is a Pareto local optimum if there exists no alignment $\phi ^\prime$ in $N(\phi )$ such that $BSP(\phi ^\prime ) \ge BSP(\phi )$.

Pareto local search (PLS) [20] is a generic local search framework for multiobjective optimization problems that follows the principle above by keeping the best alignments into a special data structure, known as the *archive*. Each neighboring alignment that is non-dominated with respect to the alignments in the archive, is added to it. The algorithm “naturally” terminates once there is no neighbor of any alignment in the archive that is not dominated (a local optimal set). See [20, 21] for more details about this approach for multiobjective combinatorial optimization problems and its successful application to scheduling and graph problems.

In the following we describe some options that were considered to apply PLS to MMSA: neighborhood function and starting alignment.

#### Neighborhood

We consider a *k*-*block* neighborhood for this problem, which consists of exchanging a substring of at most *k* characters in $\Sigma$ with indels in a *gap*, that is, a contiguous sequence of indels. In the following, we establish the conditions for two alignments to be *k*-block neighbors. Let $A_1 = (a_{1,1}, \ldots , a_{1,n_1} )$, ..., $A_m = (a_{m,1} ,\ldots , a_{m,n_m})$ denote *m* strings and let $\phi _B$ and $\phi _C$ be two alignments, where $\phi _B = B_1,\ldots , B_m$ and $\phi _C = C_1,\ldots , C_m$. The alignments $\phi _B$ and $\phi _C$ are *k*-block neighbors if and only if the following conditions hold:

(i) $$|B_i |= |C_i |$$, for $$i= 1,\ldots ,m$$;
(ii) Let $J = \{ j \ | \ j \in \{1,\ldots ,m\} : B_j \ne C_j \}$; then $|J |= 1$;
(iii) For $j \in J$ let $\pi _{B_j}^i$ and $\pi _{C_j}^i$ denote the position of $a_{j,i} \in A_j$ in string $B_j$ and $C_j$, respectively. Let $I_{B_j} = \{\pi _{B_j}^i \mid \pi _{B_j}^i \ne \pi _{C_j}^i, i=1,\ldots ,|A_j |\}$ and $I_{C_j} = \{\pi _{C_j}^i \mid \pi _{B_j}^i \ne \pi _{C_j}^i, i=1,\ldots ,|A_j |\}$. Then, $\ell = |I_{B_j} |= |I_{C_j}|\le k$.
(iv) Let $I_{B_j} = \{ I_{B_j,1},\ldots , I_{B_j,\ell }\}$ and $I_{C_j} = \{ I_{C_j,1},\ldots , I_{C_j,\ell }\}$; then $I_{B_j,i+1} - I_{B_j,i} = I_{C_j,i+1} - I_{C_j,i} = 1$, for $i=1,\ldots , \ell -1$.

Conditions (i) and (ii) state that both alignments should have the same size, and differ only in the *j*th string, respectively. In addition, condition (iii) and (iv) state that at most *k* characters from string $A_j$ do not occupy the same position in both alignments, and that those characters are contiguous. For illustration purpose, consider the following alignment:
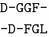


We list all possible 2-block neighbors as follows:
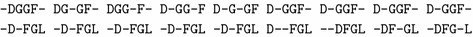


It is possible to visit all *k*-block neighbors of an alignment $\phi$ in a straightforward way. Given the first string of an alignment $\phi$, consider the leftmost substring of size one that contains only a character and an indel to its right. Then, exchange it with every indel in its right and stop when no indel is found. In case the substring has also indels to its left, repeat the same exchange procedure. If it is still possible, increase the size of the substring by one and repeat the same moves to its right and to its left; repeat the overall procedure until reaching a substring of size *k*. Then, consider the next substring of size one to the right and repeat the same procedure as above. Note that each move generates a new *k*-block neighbor of alignment $\phi$. In order to maintain feasibility, the columns that contain only indels are deleted from the alignment.

#### Starting alignment

The starting solution may have a strong impact on the overall performance of the local search. We considered the three following possibilities for PLS:

(i) Rand: A random feasible alignment, which is obtained by inserting indels randomly into the strings, except in the largest one. This initialization option tends to generate a feasible alignment with a small number of indels.
(ii) Clust: A feasible alignment obtained from program Clustal Omega [22] (available at http://www.clustal.org/omega).
(iii) Tcoffee: A feasible alignment obtained from program Tcoffee [23] (available at http://www.tcoffee.org/Projects/tcoffee/).

We choose Tcoffee since it is one of the best consistency based methods that outperforms the others existing programs in terms of accuracy and Clustal Omega since it outperforms Tcoffee whenever sequences with large N/C terminal extensions exist [24]. We used the default parameters suggested for these two programs.
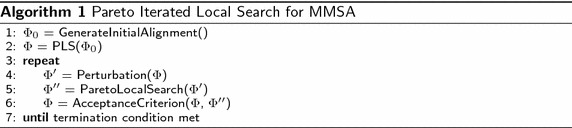


### Pareto iterated local search

The local search framework described in the previous section may get trapped in a local optima set of poor quality. One possibility for escaping from local optima is to *perturb* one or more alignments in the archive and restart the local search from those alignments. Pareto iterated local search (PILS) implements this search behavior and its main steps are presented in Algorithm 1. Similar to the single objective counterpart (see [25]), four components have to be specified in PILS:

(i) GenerateInitialSolution, which generates an initial set of alignments $\Phi _0$; based on the experimental results, we consider two starting solutions previously described (Tcoffee and Clust).
(ii) Perturbation, which modifies some of the current alignments in set $\Phi$ leading to some intermediate set of alignments $\Phi ^\prime$. Based on preliminary experiments, we decided to randomly choose 5 alignments from set $\Phi$ in such a way that the corresponding non-dominated scores are spread enough in the objective space. Then, a gap of a given size is inserted into each sequence in random positions.
(iii) PLS, which is applied to $\Phi ^\prime$ and returns an improved set of alignments $\Phi ^{\prime \prime }$.
(iv) AcceptanceCriterion, which consists of merging the two sets, $\Phi$ and $\Phi ^{\prime \prime }$, and filtering out the dominated scores.

## Discussion and results

We performed several experiments in order to understand the effect of different parameters of PLS and PILS and to establish functional relationships between algorithm performance and instance features, such as the number of sequences and their sizes. Furthermore, we compare the quality of the alignments produced by our approaches with those produced by TCoffee and Clustal Omega.

The implementations were coded in C and compiled with GCC version 4.6.1 with the −O3 compiler option. Experiments were performed in a cluster with 16 nodes, each one comprising an Intel Core i7 CPU with 4-core and 2 GB Ram, with operating system Ubuntu 11.10. Except the compiler option, no other code optimization technique was used in the experiments. For this experimental analysis, we have chosen sequences obtained from the benchmark database BAliBASE 3.0 [26]. In our substitution matrix, each cell value is 1 when the both residues are equal, and −1 otherwise.

BAliBASE 3.0 is a well-known benchmark for evaluating the multiple sequence alignment algorithms. The data set Reference 1 consists of equidistant family sequences with two subgroups: RV11 and RV12. RV11 contains 38 data sets with less than 20 % residue identity between groups and RV12 contains 44 data sets with residue identity between 20 and 40 %. Reference 2 (RV20) contains 41 alignments comprising family sequences with more than 40 % similarity and a highly divergent orphan sequence. Reference 3, with 30 data sets, contains subfamilies such that the sequences within a given subfamily share more than 40 % identity, but any two sequences from different subfamilies share less than 20 % identity. The reference set RV40, with 49 data sets, contains sequences that are composed of groups with N/C-terminal extensions. The reference set RV50 contains 16 data sets with large internal insertions. We tested our approaches on all the instances from the sets RV11 and RV20, composed by sequences with different sizes and varying percentage identities.

Since the heuristics proposed in this paper are stochastic, we ran each variant 10 times for each instance and recorded the contents of the archive for each run, namely the *approximate set*, once the termination criterion was met. To evaluate the quality of each approximate set, we computed its hypervolume indicator value. This indicator measures the area that is dominated by the approximate set, bounded by a reference point [27]; see an in-depth discussion about the hypervolume indicator in relation with other performance assessment methods in Zitzler et al. [28]. Moreover, it is known that the hypervolume is maximized when the optimal set is found [28].

**Fig. 1:**
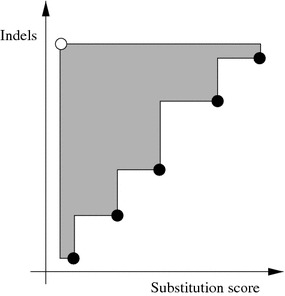
Illustration of the hypervolume indicator [17]

Figure 1 illustrates the hypervolume indicator (shaded area) for a given approximate set (black points) and a given reference point (white point). We have chosen, as the reference point for each instance, the minimum substitution score minus one and the maximum number of indels plus one that were obtained from the runs of all variants. We merged all approximate sets produced from all runs for a given instance, extracted the non-dominated scores and computed the hypervolume indicator value of the resulting set, namely, the *reference hypervolume indicator value*, which is then used as a reference value to evaluate the relative performance of each approach. Then, for each approximate set, we computed its hypervolume indicator value and the percentage of this value with respect to the reference hypervolume indicator value; the larger the value, the better is the approximate set in terms of this indicator.

We analysed the effect of different *k*-values in the *k*-block neighborhood, as well as the effect of the three starting alignments on the performance of PLS. For a given instance, let Min denote the length of the smallest string; we consider $k \in \{\mathtt{Min},\mathtt{Min}/2,\mathtt{Min}/4,\mathtt{Min}/8,\mathtt{Min}/16\}$. PLS terminates once it is not possible to find non-dominated neighboring alignments or the time limit of 5 minutes of CPU-time is reached. Tables 1 and 2 report the results obtained for PLS for the benchmark sets RV11 and RV20. The results were averaged over 10 runs, for each of the five *k* values and the three starting alignments. Column id corresponds to the instance id from the two benchmark sets (BB1100*.tfa and BB200*.tfa, where * denotes the id); column *m* gives the number of sequences; columns Min and Max correspond to the length of the smallest and the largest sequence, respectively. The instances are lexicographically ordered in terms of the number of sequences and the length of the smallest sequence. The values in italics correspond to the best result obtained for each instance. The last column Init shows the hypervolume indicator obtained for the non-dominated score of the two starting alignments obtained with Clustal Omega and Tcoffee.

**Table 1:** Percentage with respect to the reference hypervolume indicator value for local search with different *k*-block neighborhood sizes and three starting alignments (Clustal Omega, Tcoffee and Rand) for each benchmark instance in dataset RV11. The results are averaged over 10 runs. See text for more details

				$k = \mathtt{Min}$			$k = \mathtt{Min}/2$			$k = \mathtt{Min}/4$			$k = \mathtt{Min}/8$			$k = \mathtt{Min}/16$			
id	*m*	Min	Max	Rand	Clust	Tcoff	Rand	Clust	Tcoff	Rand	Clust	Tcoff	Rand	Clust	Tcoff	Rand	Clust	Tcoff	Init
22	4	63	205	0.86	0.77	0.90	0.81	0.67	*0.90*	0.86	0.64	0.89	0.83	0.52	0.71	0.72	0.45	0.55	0.14
25	4	64	103	0.53	0.84	0.84	0.54	0.76	0.86	0.51	0.72	*0.86*	0.53	0.64	0.74	0.48	0.55	0.49	0.28
29	4	81	138	0.69	0.81	0.85	0.67	0.85	*0.88*	0.69	0.85	0.87	0.65	0.82	0.85	0.65	0.72	0.81	0.35
1	4	83	91	0.17	0.27	0.35	0.17	0.25	*0.35*	0.16	0.24	0.34	0.13	0.26	0.34	0.10	0.28	0.33	0.20
9	4	97	337	0.61	*0.87*	0.78	0.61	0.71	0.67	0.60	0.51	0.44	0.59	0.40	0.22	0.53	0.30	0.10	0.19
21	4	102	139	0.51	*0.91*	0.86	0.54	0.86	0.90	0.53	0.78	0.81	0.54	0.72	0.76	0.52	0.61	0.72	0.18
8	4	104	540	0.79	*0.89*	0.84	0.79	0.89	0.82	0.79	0.76	0.86	0.80	0.49	0.81	0.74	0.33	0.75	0.03
17	4	247	264	0.37	0.90	0.88	0.33	0.90	*0.91*	0.33	0.85	0.88	0.31	0.82	0.84	0.27	0.74	0.78	0.36
15	4	297	327	0.46	0.92	0.87	0.44	0.93	0.89	0.53	0.89	0.93	0.49	0.88	*0.94*	0.45	0.90	0.92	0.38
12	4	320	397	0.38	0.91	0.87	0.36	*0.92*	0.90	0.44	0.91	0.90	0.36	0.87	0.87	0.36	0.84	0.82	0.33
24	4	372	465	0.50	0.89	0.83	0.50	*0.90*	0.90	0.50	0.80	0.76	0.52	0.74	0.73	0.51	0.66	0.68	0.17
4	4	390	456	0.35	*0.91*	0.85	0.36	*0.91*	0.88	0.36	0.90	0.89	0.35	0.84	0.85	0.35	0.76	0.77	0.23
3	4	414	516	0.43	*0.92*	0.88	0.42	0.90	0.90	0.41	0.89	0.90	0.42	0.86	0.90	0.42	0.84	0.88	0.38
10	4	490	492	0.03	*0.95*	0.85	0.03	0.91	0.84	0.03	0.80	0.82	0.01	0.72	0.80	0.01	0.67	0.73	0.25
13	5	51	101	0.69	0.80	0.83	0.69	0.81	*0.87*	0.69	0.72	0.83	0.66	0.70	0.80	0.63	0.58	0.67	0.06
35	5	71	138	0.72	0.84	0.81	0.71	0.85	0.79	0.74	*0.85*	0.80	0.74	0.76	0.79	0.67	0.62	0.77	0.14
11	5	160	242	0.54	*0.94*	0.87	0.54	0.94	0.90	0.52	0.92	0.90	0.50	0.80	0.85	0.53	0.69	0.76	0.10
37	5	335	1192	0.38	0.68	0.67	0.46	0.73	0.74	0.54	0.79	0.81	0.62	0.80	0.88	0.70	0.81	*0.93*	0.24
14	6	502	634	0.39	0.90	0.85	0.36	0.93	0.90	0.47	0.94	0.94	0.37	*0.96*	0.96	0.50	0.95	0.95	0.52
26	7	76	906	0.61	0.75	0.77	0.76	0.79	0.85	0.88	0.84	0.91	0.89	0.79	0.92	*0.96*	0.66	0.82	0.13
27	7	175	432	0.48	0.82	0.72	0.57	0.88	0.78	0.65	0.94	0.83	0.71	*0.95*	0.84	0.68	0.91	0.79	0.24
23	7	231	407	0.54	0.81	0.76	0.61	0.86	0.81	0.66	0.90	0.88	0.62	*0.93*	0.89	0.72	0.91	0.83	0.37
2	8	52	193	0.85	0.87	*0.88*	0.84	0.86	0.87	0.85	0.82	0.86	0.84	0.73	0.83	0.82	0.67	0.78	0.26
6	8	186	283	0.31	0.80	0.73	0.31	0.86	0.77	0.32	0.86	0.83	0.32	*0.88*	*0.88*	0.33	0.80	0.86	0.28
32	8	226	403	0.45	0.80	0.76	0.49	0.85	0.82	0.55	0.88	0.86	0.57	0.92	0.88	0.60	*0.93*	0.90	0.40
38	8	261	614	0.23	0.79	0.76	0.28	0.82	0.80	0.35	0.89	0.86	0.47	0.90	0.92	0.54	0.91	*0.94*	0.37
36	8	298	436	0.38	0.78	0.68	0.43	0.83	0.73	0.44	0.87	0.79	0.49	0.92	0.85	0.48	*0.94*	0.87	0.41
16	8	316	729	0.27	0.71	0.77	0.32	0.76	0.82	0.38	0.80	0.85	0.50	0.83	0.89	0.58	0.88	*0.95*	0.37
34	8	401	729	0.27	0.69	0.79	0.30	0.72	0.83	0.36	0.75	0.85	0.46	0.79	0.89	0.54	0.81	*0.94*	0.32
20	9	201	237	0.29	0.87	0.76	0.30	0.90	0.83	0.30	*0.92*	0.87	0.27	0.89	0.88	0.27	0.86	0.89	0.40
7	9	385	457	0.37	0.79	0.68	0.39	0.84	0.77	0.38	0.88	0.80	0.41	0.92	0.89	0.41	*0.93*	0.89	0.37
28	10	93	211	0.53	0.90	0.85	0.56	*0.91*	0.90	0.56	0.86	0.89	0.56	0.84	0.86	0.51	0.77	0.82	0.40
19	10	299	396	0.35	0.74	0.74	0.38	0.80	0.76	0.40	0.85	0.82	0.42	*0.91*	0.87	0.44	*0.91*	0.90	0.43
33	11	85	239	0.57	0.78	0.82	0.62	0.82	0.83	0.64	0.83	0.89	0.67	0.80	*0.92*	0.66	0.75	0.88	0.26
31	11	300	611	0.22	0.77	0.73	0.26	0.80	0.75	0.31	0.85	0.77	0.40	0.88	0.84	0.47	*0.96*	0.84	0.21
30	14	236	392	0.23	0.73	0.76	0.28	0.77	0.76	0.31	0.81	0.80	0.37	0.87	0.85	0.40	*0.90*	0.89	0.51
5	14	329	465	0.20	0.70	0.71	0.21	0.73	0.73	0.26	0.75	0.76	0.30	0.82	0.79	0.31	*0.85*	*0.85*	0.53
18	14	418	750	0.09	0.71	0.87	0.12	0.73	0.88	0.15	0.76	0.87	0.19	0.79	0.88	0.24	0.82	*0.90*	0.45

**Table 2:** Percentage with respect to the reference hypervolume indicator value for local search for different *k*-block neighborhood sizes and starting solutions (Clustal Omega, Tcoffee and Rand) for each benchmark instancein dataset RV20. The results are averaged over 10 runs. See text for more details

				$k = \mathtt{Min}$			$k = \mathtt{Min}/2$			$k = \mathtt{Min}/4$			$k = \mathtt{Min}/8$			$k = \mathtt{Min}/16$			
id	*m*	Min	Max	Clust	Tcoff	Rand	Clust	Tcoff	Rand	Clust	Tcoff	Rand	Clust	Tcoff	Rand	Clust	Tcoff	Rand	Init
20	16	74	697	0.73	0.90	0.27	0.72	*0.90*	0.28	0.68	0.86	0.34	0.60	0.86	0.29	0.54	0.82	0.34	0.27
1	16	247	527	0.95	0.60	0.16	*0.95*	0.56	0.19	0.88	0.53	0.22	0.82	0.47	0.18	0.73	0.34	0.17	0.50
2	20	52	1520	0.82	0.50	0.65	*0.82*	0.49	0.67	0.80	0.52	0.69	0.76	0.45	0.66	0.72	0.35	0.68	0.55
11	21	95	631	0.89	*0.96*	0.72	0.87	0.81	0.74	0.82	0.77	0.77	0.79	0.74	0.76	0.80	0.67	0.90	0.27
7	23	381	457	0.91	0.92	0.50	0.93	*0.93*	0.50	0.91	0.93	0.55	0.84	0.89	0.53	0.81	0.82	0.66	0.23
19	24	401	729	0.87	0.93	0.62	0.94	0.95	0.67	0.91	0.96	0.65	0.86	*0.96*	0.66	0.83	0.87	0.75	0.42
16	27	106	458	*0.93*	0.89	0.69	0.85	0.88	0.73	0.74	0.80	0.78	0.67	0.78	0.72	0.63	0.72	0.72	0.46
12	27	210	634	*0.95*	0.75	0.35	0.89	0.75	0.37	0.77	0.71	0.37	0.50	0.59	0.36	0.39	0.52	0.42	0.12
13	28	447	747	*0.70*	0.72	0.25	0.83	0.77	0.30	0.85	0.83	0.26	0.79	0.86	0.30	0.63	0.94	0.32	0.08
29	29	64	167	0.76	*0.87*	0.74	0.67	0.83	0.78	0.66	0.80	0.77	0.56	0.82	0.74	0.53	0.79	0.78	0.25
9	29	254	415	0.87	0.88	0.22	*0.94*	0.92	0.26	0.93	0.94	0.30	0.82	0.75	0.26	0.74	0.67	0.23	0.48
27	29	279	1052	0.75	0.62	0.28	0.83	0.70	0.31	0.84	0.76	0.36	*0.88*	0.83	0.32	0.87	0.87	0.45	0.35
10	29	577	1233	0.51	0.74	0.26	0.64	0.80	0.31	0.85	0.87	0.29	0.90	*0.96*	0.31	0.92	0.88	0.44	0.05
24	30	74	739	0.81	0.65	0.34	0.83	0.79	0.34	0.86	0.82	0.41	0.85	*0.90*	0.36	0.81	0.84	0.39	0.31
23	31	202	244	*0.96*	0.89	0.31	0.92	0.89	0.36	0.91	0.86	0.32	0.86	0.81	0.35	0.71	0.71	0.43	0.37
26	32	271	1016	0.78	0.64	0.44	0.84	0.73	0.47	0.85	0.83	0.45	*0.87*	0.86	0.49	0.76	0.85	0.59	0.34
35	35	226	982	0.69	0.83	0.24	0.73	0.64	0.27	0.76	0.68	0.32	*0.85*	0.72	0.24	0.81	0.73	0.37	0.25
15	37	54	274	0.71	0.85	0.77	0.80	0.94	0.80	0.58	*0.94*	0.83	0.48	0.86	0.81	0.45	0.80	0.79	0.03
38	42	79	199	0.91	*0.95*	0.73	0.87	0.94	0.76	0.76	0.84	0.81	0.65	0.74	0.74	0.62	0.69	0.84	0.52
5	42	343	474	0.49	0.87	0.43	0.57	0.91	0.44	0.63	0.93	0.50	0.65	*0.94*	0.47	0.64	0.97	0.60	0.11
17	45	509	713	0.54	0.76	0.74	0.62	0.81	0.78	0.69	0.89	0.74	0.81	0.97	0.74	0.75	*0.99*	0.76	0.31
30	47	76	155	0.49	0.84	0.18	0.55	*0.88*	0.20	0.54	0.83	0.27	0.52	0.76	0.19	0.51	0.83	0.27	0.30
33	48	81	155	*0.93*	0.87	0.57	0.89	0.82	0.58	0.78	0.79	0.64	0.66	0.67	0.61	0.65	0.69	0.59	0.57
41	48	293	1520	*0.71*	0.40	0.02	0.64	0.42	0.07	0.61	0.44	0.06	0.59	0.48	0.02	0.60	0.53	0.07	0.39
6	51	224	293	0.74	0.93	0.26	0.78	0.95	0.31	0.69	*0.96*	0.30	0.69	0.93	0.27	0.68	0.86	0.43	0.15
18	53	296	381	0.86	0.71	0.31	*0.99*	0.89	0.31	0.94	0.96	0.31	0.82	0.84	0.34	0.67	0.67	0.34	0.46
21	53	418	838	0.76	0.65	0.27	0.75	0.72	0.33	0.78	0.77	0.28	0.74	*0.81*	0.32	0.72	0.83	0.30	0.36
28	54	241	610	0.77	0.97	0.72	0.79	0.97	0.72	0.83	*0.97*	0.79	0.88	0.93	0.75	0.90	0.91	0.78	0.30
4	55	401	734	0.80	0.72	0.21	0.76	0.76	0.25	0.80	0.80	0.25	0.87	*0.89*	0.24	0.87	0.93	0.39	0.19
8	56	98	1520	0.60	0.40	0.43	0.61	0.47	0.46	0.60	0.57	0.45	0.61	*0.68*	0.46	0.61	0.72	0.48	0.16
22	58	320	381	0.67	0.63	0.11	0.74	0.68	0.15	0.84	0.78	0.17	0.89	0.87	0.13	0.88	*0.91*	0.29	0.39
34	59	234	548	0.60	0.78	0.57	0.68	0.79	0.62	0.75	0.83	0.60	0.81	0.85	0.61	0.79	*0.89*	0.64	0.33
31	60	175	432	0.81	0.71	0.40	0.79	0.80	0.45	0.74	0.86	0.47	0.65	0.92	0.40	0.61	*0.89*	0.58	0.38
32	61	93	149	0.97	0.92	0.48	*0.98*	0.90	0.52	0.78	0.85	0.57	0.67	0.72	0.52	0.63	0.67	0.58	0.35
37	65	160	810	0.65	0.49	0.03	0.59	0.54	0.04	0.56	0.59	0.05	0.56	0.66	0.08	0.55	*0.73*	0.13	0.42
14	65	333	729	0.62	0.83	0.43	0.67	0.83	0.48	0.63	0.84	0.45	0.62	0.82	0.46	0.69	*0.90*	0.46	0.54
3	74	409	800	0.52	0.71	0.77	0.69	0.88	0.81	0.60	0.80	0.84	0.54	0.75	0.80	0.73	*0.93*	0.83	0.60
25	81	372	518	0.56	0.84	0.20	0.45	0.87	0.21	0.49	0.86	0.30	0.48	0.83	0.25	0.43	*0.99*	0.26	0.36
40	87	273	570	0.44	0.77	0.33	0.36	0.87	0.34	0.38	0.86	0.34	0.41	0.83	0.36	0.36	*0.90*	0.51	0.11
36	91	82	335	0.61	0.79	0.22	0.74	0.78	0.27	0.68	0.79	0.28	0.61	*0.85*	0.26	0.81	0.78	0.38	0.18
39	91	298	458	0.52	0.85	0.20	0.40	0.82	0.22	0.45	0.85	0.22	0.49	*0.94*	0.22	0.39	0.76	0.29	0.33

The results clearly indicate the best performance of PLS when starting with alignments Clust and Tcoffee, instead of a feasible random alignment. Also, the best *k*-block value strongly depends on the number of sequences and, to a smaller extent, to their sizes. As a rule, for the given cut-off time, large (small) *k* values achieve better performance on problems with a smaller (larger) number of sequences. Moreover, column Init indicates that PLS strongly improves upon the two starting alignments.

Tables 3 and 4 report results obtained for PILS on the same benchmark sets, averaged over 10 runs, for *k*-block size equals to 2; we recall that we use Clust and Tcoffee as starting alignments. The results reported in the tables do not include the CPU-time taken to compute the starting alignments. PILS always terminates once the time limit of 5 minutes of CPU-time is reached. In this case, the limit time for each PLS run within PILS is $5/(P+1)$ minutes. Column $\mathtt{PLS}^{max}$ gives the best value of PLS from Tables 1 and 2. The best results for each sequence set are shown in italics. In most of the instances, PILS improves over PLS, although the best performance may depend on the number of sequences and their size: a small (large) number of perturbations gives better performance on larger (smaller) sequences and larger (smaller) number of sequences.

**Table 3:** Percentage with respect to the reference hypervolume indicator value for PILS with several perturbation number (P) for each instances of data set RV11 [17]. The results are averaged over 10 runs. See text for more details

id	*m*	Min	Max	$\mathtt{PLS}^{max}$	P = 1	P = 2	P = 4	P = 8	P = 16
22	4	63	205	0.90	0.91	0.87	0.89	0.88	*0.97*
25	4	64	103	0.86	0.86	0.89	*0.91*	0.89	0.87
29	4	81	138	0.88	0.91	0.91	0.94	0.92	*0.98*
1	4	83	91	0.35	0.36	0.38	0.40	0.42	*0.44*
9	4	97	337	0.87	0.87	0.81	*0.87*	0.83	0.86
21	4	102	139	0.91	*0.95*	0.89	0.92	0.88	0.93
8	4	104	540	0.89	0.87	0.91	*0.97*	0.88	0.90
17	4	247	264	0.91	0.92	0.93	0.95	0.96	*0.97*
15	4	297	327	0.94	0.90	0.92	0.96	*0.97*	*0.97*
12	4	320	397	0.92	0.92	0.91	0.96	0.97	*0.99*
24	4	372	465	*0.90*	0.87	0.85	0.86	0.83	0.80
4	4	390	456	0.91	*0.93*	0.87	0.88	0.88	0.84
3	4	414	516	0.92	*0.92*	0.89	0.89	0.88	0.88
10	4	490	492	0.95	*0.97*	0.95	0.96	0.90	0.94
13	5	51	101	0.87	0.91	0.92	0.93	0.93	*0.95*
35	5	71	138	0.85	0.86	0.89	0.88	0.93	*0.99*
11	5	160	242	*0.94*	*0.94*	0.89	0.91	0.88	0.91
37	5	335	1192	0.93	0.92	0.94	0.91	*0.99*	0.98
14	6	502	634	0.96	0.95	*0.97*	0.95	0.94	0.91
26	7	76	906	0.96	*0.99*	0.96	0.95	0.66	0.60
27	7	175	432	*0.95*	0.91	0.87	0.85	0.85	0.69
23	7	231	407	*0.93*	0.89	0.81	0.82	0.82	0.79
2	8	52	193	0.88	*0.92*	0.84	0.82	0.84	0.78
6	8	186	283	*0.88*	*0.88*	0.77	0.75	0.75	0.66
32	8	226	403	*0.93*	0.88	0.85	0.83	0.81	0.74
38	8	261	614	*0.94*	0.86	0.88	0.89	0.85	0.81
36	8	298	436	0.94	0.94	0.94	0.94	0.96	*0.97*
16	8	316	729	0.95	0.94	0.93	0.96	0.95	*0.99*
34	8	401	729	*0.94*	0.85	0.83	0.81	0.80	0.77
20	9	201	237	0.92	0.96	*0.97*	0.96	0.90	0.83
7	9	385	457	0.93	0.92	0.92	*0.97*	0.93	0.88
28	10	93	211	0.91	*0.95*	*0.95*	0.90	0.87	0.86
19	10	299	396	*0.91*	0.85	0.85	0.83	0.83	0.81
33	11	85	239	*0.92*	*0.92*	0.91	0.90	0.87	0.84
31	11	300	611	*0.96*	0.95	0.88	0.89	0.84	0.82
30	14	236	392	*0.90*	0.89	0.86	0.81	0.80	0.78
5	14	329	465	0.85	*0.87*	0.83	0.80	0.75	0.75
18	14	418	750	0.90	*0.94*	0.92	0.90	0.88	0.87

**Table 4:** Percentage with respect to the reference hypervolume indicator value for PILS with several perturbation number (P) for each instances of data set RV20. The results are averaged over 10 runs. See text for more details

id	*m*	Min	Max	$\mathtt{PLS}^{max}$	P = 1	P = 2	P = 4	P = 8	P = 16
20	16	74	697	*0.95*	*0.95*	*0.95*	0.88	0.83	0.73
1	16	247	527	*0.90*	*0.90*	*0.90*	0.87	0.88	0.85
2	20	52	1520	0.82	0.93	*0.94*	*0.94*	0.89	0.84
11	21	95	631	0.90	*0.97*	0.95	0.91	0.86	0.85
7	23	381	457	0.93	0.95	*0.96*	0.95	0.89	0.83
19	24	401	729	*0.96*	0.91	0.94	0.95	0.95	0.87
16	27	106	458	0.89	*0.91*	0.90	0.80	0.78	0.72
12	27	210	634	*0.89*	*0.89*	0.82	0.80	0.59	0.52
13	28	447	747	*0.94*	0.69	0.77	0.86	0.83	0.87
29	29	64	167	0.83	*0.89*	0.84	0.82	0.81	0.81
9	29	254	415	0.94	0.89	*0.96*	0.94	0.82	0.76
27	29	279	1052	0.87	0.73	0.81	0.87	0.90	*0.92*
10	29	577	1233	0.92	0.64	0.73	0.80	0.90	*0.94*
24	30	74	739	0.86	0.80	0.85	0.90	*0.91*	0.88
23	31	202	244	0.92	*0.98*	0.95	0.91	0.87	0.73
26	32	271	1016	0.86	0.73	0.85	0.90	*0.93*	0.82
35	35	226	982	0.83	0.80	0.78	0.82	0.88	*0.90*
15	37	54	274	*0.94*	0.82	0.91	*0.94*	0.86	0.80
38	42	79	199	*0.94*	*0.94*	*0.94*	0.84	0.73	0.69
5	42	343	474	*0.97*	0.83	0.85	0.87	0.91	0.93
17	45	509	713	*0.97*	0.71	0.74	0.81	0.87	0.94
30	47	76	155	0.84	0.84	*0.87*	0.83	0.80	0.83
33	48	81	155	0.89	*0.95*	0.89	0.81	0.67	0.69
41	48	293	1520	0.64	0.88	0.84	0.82	0.83	*0.86*
6	51	224	293	*0.95*	0.85	0.91	*0.95*	0.93	0.86
18	53	296	381	*0.96*	0.70	0.87	0.94	0.83	0.67
21	53	418	838	0.83	0.89	0.91	0.93	*0.94*	0.91
28	54	241	610	*0.97*	0.96	*0.97*	0.96	0.94	0.93
4	55	401	734	0.93	0.81	0.80	0.84	0.89	*0.94*
8	56	98	1520	0.72	0.72	0.78	0.81	0.85	*0.87*
22	58	320	381	0.89	0.62	0.68	0.79	0.91	*0.92*
34	59	234	548	0.85	0.75	0.75	0.82	*0.88*	0.86
31	60	175	432	*0.92*	0.87	0.87	0.83	0.82	0.88
32	61	93	149	0.97	0.97	*0.98*	0.88	0.73	0.67
37	65	160	810	0.84	0.80	0.84	0.81	0.80	*0.91*
14	65	333	729	0.66	0.84	0.84	0.83	0.86	*0.90*
3	74	409	800	0.88	0.73	0.90	0.81	0.76	*0.95*
25	81	372	518	0.87	0.82	0.86	0.85	0.81	*0.96*
40	87	273	570	0.87	0.83	0.92	0.91	0.88	*0.94*
36	91	82	335	0.81	0.82	0.83	0.77	0.78	*0.92*
39	91	298	458	*0.85*	0.73	0.76	0.79	0.81	*0.85*

We complemented the analysis to gain insight into the absolute quality of the alignments produced by PILS, when compared to the reference alignments available in the BAliBASE benchmark. We rely on the correctly aligned pairs (SP) and columns correctly aligned (TC) measures, as performed in [15]. These ratios can be computed by the program BAliScore which is available by ftp from ftp-igbmc.u-strasbg.fr/pub/BAliBASE [29]. We used seven different data sets from BAliBASE with specific features chosen from this benchmark; see Table 5. Columns Reference and id correspond to the reference and id number of the data set in the BAliBASE benchmark; columns *m*, Min and Max correspond to the number of sequences, length of the smallest and largest sequence, respectively; Len corresponds to the size of reference alignment in the BAliBASE benchmark.

**Table 5:** The selective data sets from difference reference of the benchmark

Reference	id	Identity	*m*	Min	Max	Len
RV11	1	<20 %	4	83	91	96
RV11	4	<20 %	4	390	456	603
RV12	20	<20 and >40 %	4	118	129	141
RV12	42	<20 and >40 %	4	448	561	611
RV20	20	>40 %	16	74	697	615
RV20	1	>40 %	16	247	527	780
RV30	17	−	15	231	370	416
RV40	10	−	9	67	214	275
RV40	14	−	9	298	609	712
RV50	4	−	9	386	505	547

We ran PILS 10 times on these data sets, and for each collection of runs, we chose the best alignment produced in terms of $BSP_s$ score. We computed the SP and TC ratios by using this alignment and the reference alignment available for each chosen data set in BAliBASE. For calculation of the SP ratio, we used the substitution matrix PAM 250. Moreover, for comparison purpose, we performed the same procedure for the alignments produced by Clustal Omega and Tcoffee.

Tables 6 and 7 show the results obtained with SP and TC ratios, respectively. The italics face values represent the best ratio. From Table 6, it can be observed that PILS obtained the best value for all the tested data sets. In Table 7, the best TC ratio is either from Tcoffee or Clustal Omega. Note that a null TC value was obtained by the alignment of Clustal Omega in RV12 id 20 and RV20 id 20, and by TCoffee in set from RV20 and id 1.

**Table 6:** The results of Sp score in Tcoffee, Clustal Omega and PILS on the selected test cases

Reference	id	Clustal Omega	Tcoffee	PILS
RV11	1	0.956	0.965	*0.985*
RV11	4	0.033	0.706	*0.788*
RV12	20	0.331	0.973	*0.981*
RV12	42	0.678	0.789	*0.85*
RV20	20	0.535	0.934	*0.946*
RV20	1	0.939	0.596	*0.951*
RV30	17	0.765	0.787	*0.811*
RV40	10	0.875	0.872	*0.878*
RV40	14	0.878	0.890	*0.894*
RV50	4	0.973	0.983	*0.988*

**Table 7:** The results of TC score in Tcoffee, Clustal Omega and PILS on the selected test cases

Reference	id	Clustal Omega	Tcoffee	PILS
RV11	1	0.912	*0.930*	0.885
RV11	4	0.408	*0.554*	0.378
RV12	20	0.000	*0.957*	0.851
RV12	42	0.548	*0.662*	0.434
RV20	20	0.000	*0.775*	0.654
RV20	1	*0.775*	0.000	0.459
RV30	17	0.552	*0.581*	0.535
RV40	10	*0.639*	0.590	0.330
RV40	14	*0.671*	0.658	0.463
RV50	4	*0.919*	0.940	0.892

## Conclusions

The local search introduced in this article is able to provide high quality alignments in a reasonable amount of time. Since local search can be stuck in local optima, we propose a method that perturbs the set of alignments in the archive and restarts the local search, such as done by iterated local search. The results show that the hypervolume indicator has improved over different number of perturbations.

The size of the trade-off returned by our approaches is still considerably large, which may be overwhelming for the practitioner. One possibility is to present a smaller subset of alignments that are *representative* of the complete trade-off; such notion of representativeness may be based on metrics of uniformity (the most spread subset) or coverage (the subset that best covers the complete set). Algorithms that allow to find optimal representative subsets are available in [30].

For the future work, we might try other starting alignments that are not just based on the similarity but also taking into account other biological criteria such as the secondary structure of the alignments. Further, we may test different perturbation methods. One of the possibilities is to use the phylogenetic trees of the sequences and apply the ratchet strategy [31, 32] which may help to create a new starting solution with more information from existing results.

## References

[1] Pei J (2008). Multiple protein sequence alignment. Curr Opin Struct Biol.

[2] Gelly JC, Joseph AP, Srinivasan N, De Brevern AG (2011). iPBA: a tool for protein structure comparison using sequence alignment strategies. Nucleic Acids Res.

[3] Wang LS, Leebens Mack J, Wall PK, Beckmann K, de Pamphilis CW, Warnow T (2011). The impact of multiple protein sequence alignment on phylogenetic estimation. IEEE/ACM Trans Comput Biol Bioinform.

[4] Setlow JK (2007). Genetic engineering: principles and methods, vol. 28.

[5] Needleman SB, Wunsch CD (1970). A general method applicable to the search for similarities in the amino acid sequence of two proteins. J Mol Biol.

[6] Morrison DA (2015). Is sequence alignment an art or a science?. Syst Bot.

[7] Abbasi M, Paquete L, Liefooghe A, Pinheiro M, Matias P (2013). Improvements on bicriteria pairwise sequence alignment: algorithms and applications. Bioinformatics.

[8] DeRonne KW, Karypis G (2013). Pareto optimal pairwise sequence alignment. IEEE/ACM Trans Comput Biol Bioinform.

[9] Paquete L, Matias P, Abbasi M, Pinheiro M (2014). Mosal: software tools for multiobjective sequence alignment. Source Code Biol Med.

[10] Roytberg MA, Semionenkov MN, Tabolina OI (1999). Pareto-optimal alignment of biological sequences. Biophysics.

[11] Taneda A (2010). Multi-objective pairwise RNA sequence alignment. Bioinformatics.

[12] Schnattinger T, Schöning U, Kestler H (2013). Structural RNA alignment by multi-objective optimization. Bioinformatics.

[13] Handl J, Kell DB, Knowles J (2007). Multiobjective optimization in bioinformatics and computational biology. IEEE/ACM Trans Comput Biol Bioinform.

[14] Ortuño FM, Florido JP, Urquiza JM, Pomares H, Prieto A, Rojas I. Optimization of multiple sequence alignment methodologies using a multiobjective evolutionary algorithm based on nsga-ii. In: IEEE Congress on evolutionary computation (CEC). New York: IEEE; 2012. pp. 1–8.

[15] Ortuño FM, Valenzuela O, Rojas F, Pomares H, Florido JP, Urquiza JM, Rojas I (2013). Optimizing multiple sequence alignments using a genetic algorithm based on three objectives: structural information, non-gaps percentage and totally conserved columns. Bioinformatics.

[16] Soto W, Becerra D. A multi-objective evolutionary algorithm for improving multiple sequence alignments. In: Advances in bioinformatics and computational biology—Proceedings of the 9th Brazilian symposium on bioinformatics, BSB 2014, Belo Horizonte, Brazil, October 28–30; 2014. pp. 73–82.

[17] Abbasi M, Paquete L, Pereira FB, Ortuño F, Rojas I (2015). Local search for multiobjective multiple sequence alignment. Bioinformatics and biomedical engineering: Third International Conference, IWBBIO 2015, Granada, Spain, April 15–17, Proceedings, part II.

[18] Bockenhauer HJ, Bongartz D (2007). Algorithmic aspects of bioinformatics.

[19] Ehrgott M (2005). Multicriteria optimization.

[20] Paquete L, Schiavinotto T, Stützle T (2007). On local optima in multiobjective combinatorial optimization problems. Ann Oper Res.

[21] Liefooghe A, Humeau J, Mesmoudi S, Jourdan L, Talbi E (2012). On dominance-based multiobjective local search: design, implementation and experimental analysis on scheduling and traveling salesman problems. J Heuristics.

[22] Sievers F, Wilm A, Dineen D, Gibson TJ, Karplus K, Li W, Lopez R, McWilliam H, Remmert M, Soding J, Thompson JD, Higgins DG (2011). Fast, scalable generation of high-quality protein multiple sequence alignments using Clustal Omega. Mol Syst Biol.

[23] Notredame C, Higgins DG, Heringa J (2000). T-coffee: a novel method for fast and accurate multiple sequence alignment. J Mol Biol.

[24] Pais FS, de Ruy P, Oliveira G, Coimbra R (2014). Assessing the efficiency of multiple sequence alignment programs. Algorithms Mol Biol.

[25] Lourenço HR, Martin OC, Stützle T (2013). Iterated local search: handbook of metaheuristics. International series in operations research and management science, vol. 57.

[26] Thompson JD, Koehl P, Ripp R, Poch O, Thompson JD, Koehl P, Ripp R, Poch O (2005). BAliBASE 3.0: latest developments of the multiple sequence alignment benchmark. Proteins.

[27] Zitzler E, Thiele L. Multiobjective optimization using evolutionary algorithms—a comparative case study. Proceedings of the 5th international conference on parallel problem solving from nature, PPSN. Berlin: Springer; 1998. p. 292–304.

[28] Zitzler E, Thiele L, Laumanns M, Fonseca CM, da Fonseca V (2003). Performance assessment of multiobjective optimizers: an analysis and review. IEEE Trans Evol Comput.

[29] Thompson JD, Plewniak F, Poch O (1999). A comprehensive comparison of multiple sequence alignment programs. Nucleic Acids Res.

[30] Vaz D, Paquete L, Fonseca CM, Klamroth K, Stiglmayr M (2015). Representation of the non-dominated set in biobjective discrete optimization. Comput Oper Res.

[31] Nixon KC (1999). The parsimony ratchet, a new method for rapid parsimony analysis. Cladistics.

[32] Morrison DA (2007). Increasing the efficiency of searches for the maximum likelihood tree in a phylogenetic analysis of up to 150 nucleotide sequences. Syst Biol.

